# Recursion-Transform method to a non-regular *m*×*n* cobweb with an arbitrary longitude

**DOI:** 10.1038/srep11266

**Published:** 2015-06-15

**Authors:** Zhi-Zhong Tan

**Affiliations:** 1 Department of physics, Nantong University, Nantong, 226019, China

## Abstract

A general Recursion-Transform method is put forward and is applied to resolving a difficult problem of the two-point resistance in a non-regular *m* × *n* cobweb network with an arbitrary longitude (or call radial), which has never been solved before as the Green’s function technique and the Laplacian matrix approach are difficult in this case. Looking for the explicit solutions of non-regular lattices is important but difficult, since the non-regular condition is like a wall or trap which affects the behavior of finite network. This paper gives several general formulae of the resistance between any two nodes in a non-regular cobweb network in both finite and infinite cases by the *R*-*T* method which, is mainly composed of the characteristic roots, is simpler and can be easier to use in practice. As applications, several interesting results are deduced from a general formula, and a globe network is generalized.

The computation of the two-point resistance in a resistor network is a classical problem in circuit theory and graph theory, which has been researched for more than 160 years. The computation of resistance is relevant to a wide range of problems ranging from classical transport in disordered media^1^, first-passage processes^2^, lattice Greens functions^3^,^4^, random walks^5^, resistance distance^6^,^7^, to graph theory^4^,^8^. However, it is usually very difficult to obtain the explicit expression of resistance in a non-regular resistor networks (it means different resistors are arranged on the network, which differs from a normal resistor network), since the non-regular condition is like a wall or trap which affects the behavior of finite network. Thus the construction of and research on the models of non-regular networks therefore make sense for theories and applications.

There may be more calculation methods of equivalent resistance of a resistor network, but the main research methods which can calculate the equivalent resistance of an *m* × *n* resistor networks are only a few. For example, the first method is that Kirchhoff found the node current law (*KCL*) and the circuit voltage law (*KVL*) in 1845, in theory the methods can resolve all the problems but that is not the case because of the algebra and technique; The second method is that Cserti evaluated the two-point resistance using the lattice Green’s function^9^, his study is mainly confined to regular lattices of infinite size; The third method is Ref. 10,11 formulated a different approach (call the Laplacian matrix method), especially Ref. 11 derived the explicit expressions for the two-point resistance in arbitrary finite and infinite lattices with normative boundary (such as free, periodic boundary etc.) in terms of the eigenvalues and eigenvectors of the Laplacian matrix relies on two matrices along two vertical directions. After some improvements, the Laplacian approach has been extended to the complex impedance network^12^ and to the resistor network with zero resistor boundary^13^,^14^,^15^; The fourth method is that Tan created the Recursion-Transform (*R-T*) method^16^ (one may refer Ref. 17, 18, 19, 20, 21), which compute the equivalent resistance relies on just one matrix along one direction, and the explicit resistance is expressed by a single summation^17^,^18^,^19^,^20^,^21^. The advantage of the *R-T* method is different from the Laplacian matrix method is that it only dependent on a matrix not two matrices. Very recently Ref. 22 propose a method of nodal potentials which may be able to calculate all kinds of resistor networks, but we find it is difficult to obtain the concise expression of resistance of an arbitrary *m* × *n* resistor networks except for a series of simple resistor networks (perhaps this method will also lead to solving this problem in the future).

For the arbitrary resistor networks of various topologies, various new results of the resistor networks have been obtained by applying the *R-T* method. For example, a cobweb model^17^, a globe network^18^, a fan network^19^, a cobweb network with 2*r* boundary^20^, a hammock network^15^, especially, very recently this method was further generalized in Ref. 21, which can applied to the resistor network with an arbitrary boundary.

However, there are still some non-regular resistor networks unresolved because of the complexity of the boundary conditions of the network in real life. Thanks to the *R*-*T* method provides us with new technique to deal with the complex networks of various topologies. In this paper we will study the resistances of a complex network with arbitrary resistors on the longitude line.

Figure. 1 is called a non-regular *m* × *n* resistor cobweb which has arbitrary resistor *r*_2_ on the boundary and an arbitrary longitude with arbitrary resistors *r*_1_ (in order to facilitate our study, we refer the name in globe and call the radial as longitude, and call the circle line as latitude). This paper focus on the computation of the two-point resistance between any two nodes in the non-regular *m* × *n* cobweb network, which has never been solved before, the Green’s function technique and the Laplacian matrix approach are difficult in this case because they depend on the two matrices along two directions (one may refer the discussion at the end). Figure. 1 is a multipurpose network model, when the boundary resistor *r*_2_ = 0, the non-regular cobweb degrades into a nearly globe network as shown in Fig. 2.

## Results

The boundary resistor *r*_2_ is a key parameter since the different parameter can represent different geometric structure, such as a globe is from a nearly cobweb with *r*_2_ = 0. In this paper we will consider three cases of *r*_2_ = {0, *r*, 2*r*}, and give several results in three cases. We first define several variables 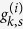, 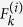, *S*_*k*,*i*_ and *t*_*i*_ for later uses by


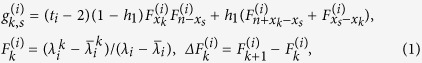



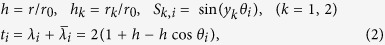


where 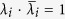, and 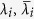 are for later uses expressed as


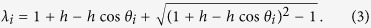


Noticing that *θ*_*i*_ has three kinds different values with three different resistors of *r*_2_ = {0, *r*, 2*r*}

### The results in the case of *r*_2_ = *r*

**Figure 1:**
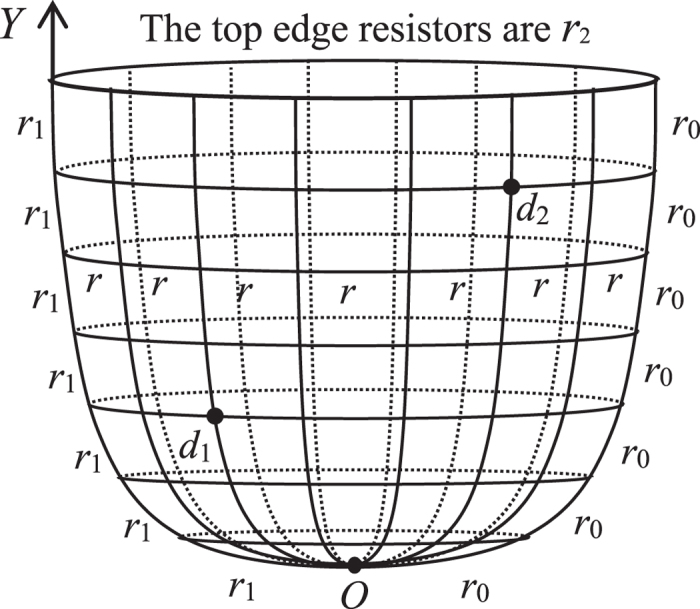
A non-regular 7 × 14 cobweb with arbitrary top boundary and an arbitrary longitude (with *r*_1_), which has 7 latitudes (including boundary) and 14 longitudes. Bonds in the longitudes and latitudes directions represent, respectively, resistors *r*_0_ and *r* except for the resistor *r*_1_ on a longitude and the boundary resistor *r*_2_.

Consider a nearly *m* × *n* resistor cobweb with resistances *r* and *r*_0_ in the respective latitudes and longitudes except for a longitude resistors *r*_1_, where *m* and *n* are, respectively, the numbers of grids along longitude and latitude directions as shown in Fig. 1. Assuming the center node *O* is the origin of the rectangular coordinate system, and a longitude with *r*_1_ act as Y axis. Denote nodes of the network by coordinate {*x*, *y*}. The equivalent resistance between any two nodes *d*_1_(*x*_1_, *y*_1_) and *d*_2_(*x*_2_, *y*_2_) in a nearly *m* × *n* cobweb network with free boundary can be written as


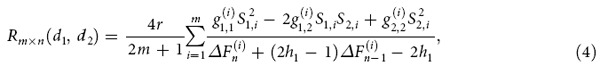


where *θ*_*i*_ = (2*i* − 1)*π*/(2*m* + 1), 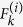, 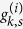 and *h*_*k*_, *S*_*k*,*i*_ are respectively defined in (1) and (2)

In particular, from (4) we have the special cases:

#### Case 1

When *h*_1_ = 1, the network degrades into a normal cobweb, we have


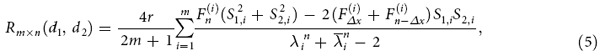


where Δ*x* = *x*_2_ − *x*_1_.

#### Case 2

When *h*_1_ = 0, we have


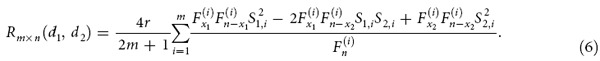


#### Case 3

When *n* → ∞, *x*_1_, *x*_2_ → ∞ with *x*_1_ − *x*_2_ finite, we have


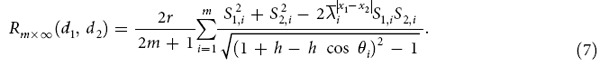


#### Case 4

When *x*_1_ = *x*_2_ = *x*, *m*,*n* → ∞ with *y*_1_, *y*_2_ finite, we have


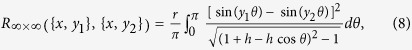


#### Case 5

When *m*,*n* → ∞, but *x*_1_ − *x*_2_ and *y*_1_ − *y*_2_ are finite, we have


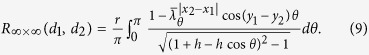


where 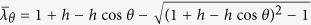.

#### Case 6

When *d*_1_ = (*x*, *y*_1_) and *d*_2_ = (*x*, *y*_2_) are both on the same longitude, we have


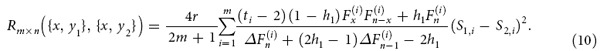


Especially, when *d*_1_ = (0, *y*_1_) and *d*_2_ = (0, *y*_2_) are both on the *Y* axis, we have


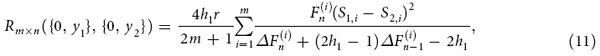


and when *n* → ∞ with *y*_1_, *y*_2_ finite, we have


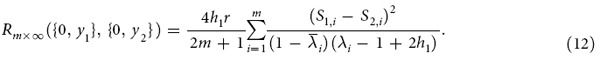


### The result in the case of *r*_2_ = 2*r*

When *r*_2_ = 2*r*, the equivalent resistance between any two nodes *d*_1_(*x*_1_, *y*_1_) and *d*_2_(*x*_2_, *y*_2_) in a nearly *m* × *n* cobweb network with 2*r* boundary is


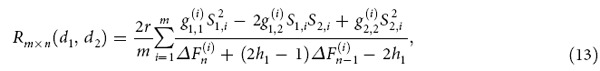


where 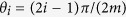. Especially, when *h*_1_ = 1, from (13) we have


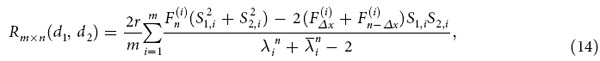


with Δ*x* = *x*_2_ − *x*_1_.

### The result in the case of *r*_2_ = 0

**Figure 2:**
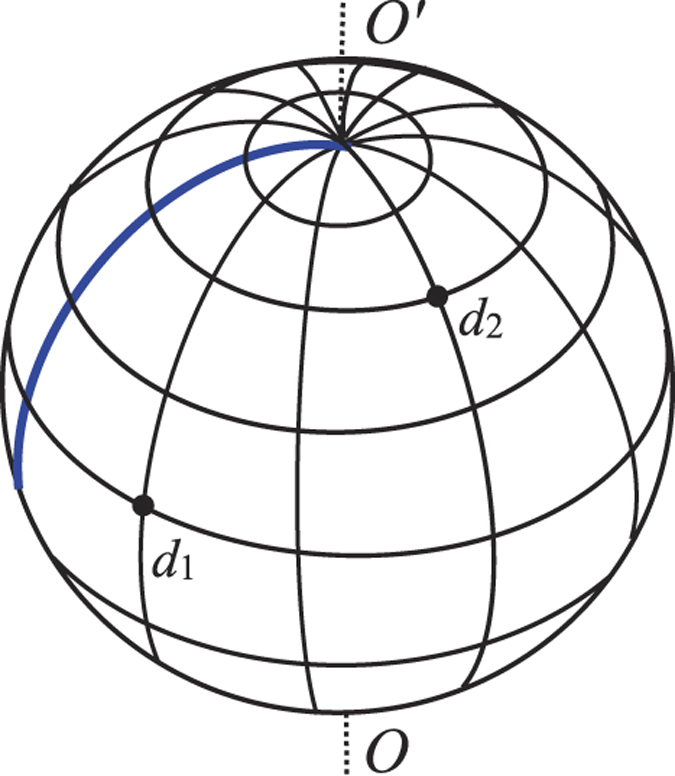
An 7 × 12 globe network with an arbitrary longitude, which has 7 latitude and 12 longitude. Bonds in the longitudes and latitudes directions represent, respectively, resistors *r*_0_ and *r* except for an arbitrary longitude.

When *r*_2_ = 0, the non-regular cobweb network degrades into a nearly globe network with an arbitrary longitude as shown in Fig. 2. The equivalent resistance between any two nodes *d*_1_(*x*_1_, *y*_1_) and *d*_2_(*x*_2_, *y*_2_) in a nearly *m* × *n* globe network with an arbitrary longitude can be written as


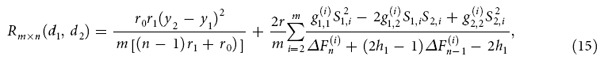


where *θ*_*k*_ = (*k* − 1)*π*/*m*. Especially, when *h*_1_ = 1, the nearly globe degrades into a normal globe network, from (15) we have


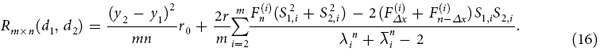


Note that: the above similar formulae are different because their *θ*_*i*_ are different from each other. The above results are original research which have not been solved before.

## Method

### Calculating resistance by Ohm’s law

**Figure 3:**
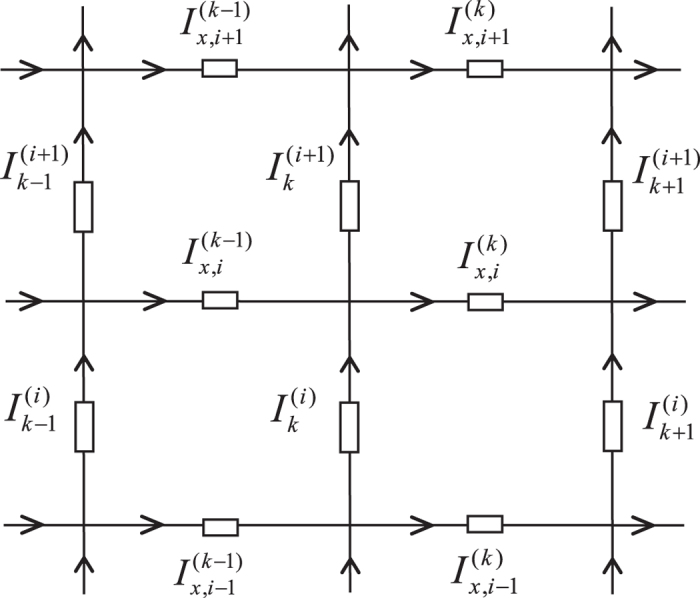
Segment of the cobweb with current parameters and directions.

Assuming the electric current *J* is constant and goes from the input *d*_1_(*x*_1_, *y*_1_) to the output *d*_2_(*x*_2_, *y*_2_) as shown in Fig. 1. Denote the currents in all segments of the network as shown in Fig. 3. The resulting currents passing through all *m* row (latitude) resistors are: 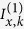, 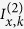, 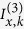
…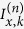 (1 ≤ *k* ≤ *m*); the resulting currents passing through all *n* column (longitude) resistors are: 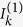, 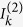,…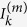 (0 ≤ *k* ≤ *n*).

To find the resistance 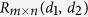, using Ohm’s law the potential difference may be measured along a path from *d*_1_(*x*_1_, *y*_1_) to *O* and then to *d*_2_(*x*_2_, *y*_2_), so we have


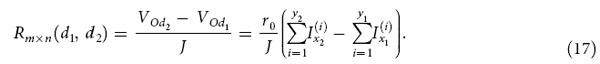


How to resolve the current parameters 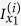 and 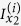 is the key to the problem. We are going to resolve the problem by the *R*-*T* approach.

### Modeling the matrix equations

A segment of the rectangular network is shown in Fig. 3. Using Kirchhoff’s law to study the resistor network, the nodes current equations and the meshes voltage equations can be achieved from Fig. 3. We focus on the four rectangular meshes and nine nodes, this gives the matrix relation (one may refer [17, 18, 19, 20, 21])


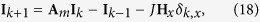


where ***I***_*k*_ and ***H***_*x*_ are respectively *m* × 1 column matrix, and reads


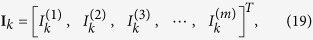



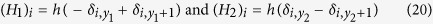


where [ ]^*T*^ denote matrix transposes, and (*H*_*k*_)_*i*_ is the elements of ***H***_*x*_ with the injection of current *J* at *d*_1_(*x*_1_, *y*_1_) and the exit of current *J* at *d*_2_(*x*_2_, *y*_2_), and ***A***_*m*_ is an *m* × *m* matrix,


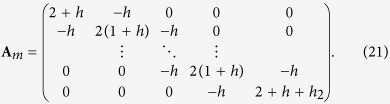


where *h* = *r*/*r*_0_, *h*_2_ = *r*_2_/*r*_0_.

We consider the bound conditions of a specific longitude with resistors *r*_1_. Applying Kirchhoff’s laws to two meshes adjacent to the specific longitude we obtain three matrix equations to model the bound currents,


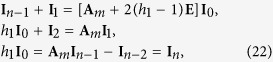


where *h*_1_ = *r*_1_/*r*_0_ and **E** is an m-dimensional identity matrix, and matrix **A**_*m*_ is given by (21).

Above Eqs.(18) ~ (22) are all equations we need to calculate the equivalent resistance of the non-regular *m* *×* *n* cobweb network with an arbitrary longitude.

### Approach of the matrix transform

To solve the matrix equation (18) we rebuild a new difference equation and resolve Eq.(18) indirectly by means of matrix transform. First, the eigenvalues *t*_*i*_ (*i* = 1, 2,…,*m*) of **A**_*m*_ are the *m* solutions of the equation


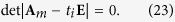


Since **A**_*m*_ is a special tridiagonal matrix which is Hermitian, it can be diagonalized by a similarity transformation to yield


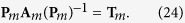


where **T**_*m*_ = diag{*t*_1_, *t*_2_,…,*t*_*m*_} is a diagonal matrix with eigenvalues *t*_*i*_ of **A**_*m*_ in the diagonal, and the column vectors of **P**_*m*_ is the eigenvector of **A**_*m*_. From (23) and (24) we obtain (2) in the cases of *r*_2_ = {0, *r*, 2*r*}, meanwhile, obtain 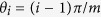, 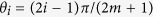 and 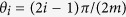, respectively, appeared in Eqs.(15), (4) and (13).

In order to implement the matrix transform, we define


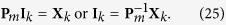


where **X**_*m*_ is an *m* × 1 column matrix, and reads


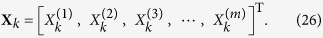


Thus we apply **P**_*m*_ to (18) on the left-hand side, and obtain a new matrix equation


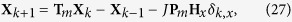


Assuming the row vectors of matrix **P**_*m*_ is


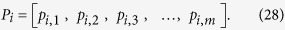


When *r*_2_ = {0, *r*, 2*r*}, we can obtain the explicit matrix **P**_*m*_. We let the *i*^th^ element of the column vector **X**_*k*_ be 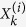. Then (27) gives


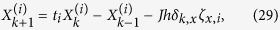


where (reads 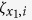
as *ζ*_1,*i*_ , and 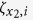
as *ζ*_2,*i*_)


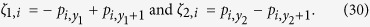


Supposing 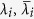 are the roots of the characteristic equation for 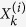, from (29) we obtain (3). Next we conduct the same matrix transform to (22), we are led to


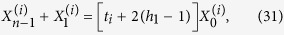



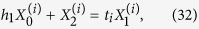



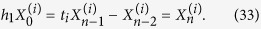


When *r*_2_ = {0, *r*, 2*r*}, from the above equations (29) ~ (33) we therefore obtain after some algebra and reduction the two solutions 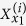 and 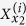 needed in our resistance calculation (17).

### Derivation of the resistance formula

According to Eq. (17), the key currents 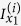 and 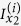 must be calculated for deriving the equivalent resistance *R*_*m* × *n*_(*d*_1_, *d*_2_). From (25) we have 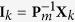, thus we obtain 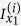 and 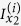. Finally, substituting 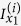 and 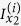 into (17), we therefore obtain the resistance *R*_*m* × *n*_(*d*_1_, *d*_2_) in three cases of *r*_2_ = {0, *r*, 2*r*}.

## Discussion

Considerable progress has recently been made in the development of techniques to exactly determine two-point resistances in the networks of various topologies. In this paper the *R*-*T* method is applied to computation of the two-point resistance in a non-regular *m* × *n* cobweb network with an arbitrary boundary and an arbitrary longitude, which has never been solved before. The resistance formulae are given in the form of a single summation, and several previous research results have become our special cases. Such as formula (5) is equivalent to the result of Ref. 19, formula (14) is the same with the result of Ref. 20, formula (16) agrees with the result of Ref. 18, and formula (9) is the same with the result of Ref. 21. Obviously, Fig. 1 is a multipurpose network model which contains several different cases.

The reason why the Green’s function technique and the Laplacian matrix approach are difficult to resolve the non-regular network in Fig. 1 is that they depend on the two matrices along two directions, besides matrix (21), there is another matrix with an arbitrary element of *r*_1_ when we set up a matrix along the latitudes directions, which is impossible for us to obtain the explicit eigenvalues and eigenvectors of a matrix with arbitrary element.

The reason why we just consider *r*_2_ = {0, *r*, 2*r*} is that, at present, we cannot obtain the explicit eigenvalues and eigenvectors of matrix (21) when *r*_2_ ≠ {0, *r*, 2*r*}, although the *R*-*T* method is feasible. Of course, we can obtain the resistance of the non-regular network as soon as we obtain the explicit eigenvalues and eigenvectors of matrix (21).

The *R*-*T* method splits the derivation into three parts. The first creates a recursion relation between the current distributions on three successive longitude lines. The second part derives a recursion relation between the current distributions on the bound longitude. The third part implements the matrix transform of diagonalization to produce a recurrence relation involving only variables on the same axis. Basically, the method reduces the problem from two dimensions to one dimension.

In addition, an important usefulness is that the *R*-*T* method can be extended to impedance networks, since the Ohm’s law based on which the method is formulated is applicable to impedances. Such as the grid elements *r* and *r*_0_ can be either a resistors or impedances, we can therefore study the *m* × *n* complex impedance networks by the *R*-*T* method.

## Additional Information

**How to cite this article**: Tan, Z.-Z. Recursion-Transform method to a non-regular *m×n* cobweb with an arbitrary longitude. *Sci. Rep.* **5**, 11266; doi: 10.1038/srep11266 (2015).
